# Chemical Prioritisation for Human Biomonitoring in Ireland: A Synergy of Global Frameworks and Local Perspectives

**DOI:** 10.3390/toxics13040281

**Published:** 2025-04-07

**Authors:** Richa Singh, Holger Martin Koch, Marike Kolossa-Gehring, Alison Connolly

**Affiliations:** 1 UCD Centre for Safety & Health at Work, School of Public Health, Physiotherapy and Sports Science, University College Dublin, D04 V1W8 Dublin, Ireland; richa.singh@ucd.ie; 2 Institute for Prevention and Occupational Medicine of the German Social Accident Insurance, Institute of the Ruhr-University Bochum (IPA), Bürkle-de-la-Camp-Platz 1, 44789 Bochum, Germany; holger.koch@dguv.de; 3 German Environment Agency (Umweltbundesamt), 06844 Dessau-Roßlau, Germany; marike.kolossa@uba.de

**Keywords:** HBM4IRE, chemical prioritisation, HBM programmes, national survey, chemicals, PARC

## Abstract

Human biomonitoring (HBM) is a critical scientific tool for assessing human exposure by quantifying chemicals and their metabolites in biological specimens such as blood and urine. This approach provides a comprehensive and accurate evaluation of internal exposures from diverse sources and exposure routes. In Ireland, establishing a national HBM programme requires a systematic chemical prioritisation process that aligns global frameworks with local public perceptions. This study integrates insights from international initiatives such as the European Joint Programme Human Biomonitoring for Europe (HBM4EU) and the Partnership for the Assessment of Risks from Chemicals (PARC)—along with HBM programmes from EU countries (Germany, France, Belgium, Norway, Slovenia, Czech Republic, and Sweden) and non-EU countries (US, Canada, South Korea, China, and New Zealand). In addition, a national survey was conducted to capture the perceptions of people in Ireland regarding chemicals of concern to develop a comprehensive priority list of chemicals and biomarkers. The broader chemical groups identified include heavy metals (lead, cadmium, mercury, arsenic, and chromium VI), plasticisers (phthalates), bisphenols, pesticides, flame retardants, PFASs (per- and polyfluoroalkyl substances), PAHs (polycyclic aromatic hydrocarbons), POPs (persistent organic compounds), VOCs (volatile organic compounds), and UV (ultraviolet) filters. This integrated, participatory approach provides a roadmap for a robust, adaptable chemical list that supports evidence-based policy decisions in HBM in Ireland and enhances public health outcomes.

## 1. Introduction

Chemicals play a fundamental role in contemporary society, driving technological advancements and economic progress. However, their high-volume use and ubiquitous presence in the environment are causing global health concerns. Studies have highlighted the adverse environmental and human health impacts associated with exposures to emerging and legacy chemicals, as well as chemical mixtures [1,2,3,4,5]. The World Health Organization (WHO) estimates that by 2030, chemical production will have doubled over the last decade, amplifying risks of human exposure and associated morbidity [6,7]. Alarmingly, 1.6 million global deaths in 2016 were attributed to chemical exposure [8], with a significant proportion linked to hazardous pollutants such as heavy metals, pesticides, and industrial chemicals, which contribute to severe health outcomes, including respiratory diseases, cancers, and neurological disorders [9,10].

To tackle the issue of chemical pollution, the European Union has adopted ambitious frameworks such as the Chemicals Strategy for Sustainability and the European Green Deal, which prioritise a “Zero Pollution Ambition” towards a toxic-free environment to ensure that chemicals are safe for health to better protect citizens and the environment against hazardous chemicals [11,12,13]. The European Environment Agency’s (EEA) ’zero pollution monitoring assessment’ identified the increasing body of evidence demonstrating that citizens’ health is being adversely affected by hazardous chemicals and that there is an urgent need to measure progress toward the zero pollution action plan’s targets and for the early detection of emerging issues [11].

Human biomonitoring (HBM) is an essential tool for evaluating chemical exposures in humans, as it analyses biological materials (e.g., blood and urine) to measure systemic availability. As HBM evaluates chemical exposures from all sources and uptake by all routes, it is a gold-standard tool for quantifying internal chemical exposures and informing evidence-based policy for zero-pollution goals [14,15,16,17,18]. By measuring contaminant uptake from air, food, and water and consumer products, HBM enables the early detection of emerging risks, evaluates regulatory efficacy, and identifies vulnerable populations [19,20]. For the past decade, initiatives like the European Joint Programme Human Biomonitoring for Europe (HBM4EU) and the Partnership for the Assessment of Risks from Chemicals (PARC) have advanced HBM through the harmonisation of protocols of biomonitoring across 30 nations, prioritising different substance groups—from phthalates to PFASs to align with EU regulatory goals [21,22,23,24,25].

The harmonised HBM studies throughout Europe have demonstrated the capabilities of HBM to evaluate chemicals of concern and to support evidence-based European policymaking [26,27]. The World Health Organization (WHO) recognises and promotes the use of HBM as an effective instrument to support policies and actions on chemical safety [28]. The WHO Regional Office for Europe (WHO/Europe has developed the “Human Biomonitoring. Basics: educational course” to build capacity for planning and implementing national HBM programmes, which cover scientific principles, practical case studies, and all stages of HBM surveys [29]. The WHO is also promoting the establishment of HBM programmes as a recognised priority for chemical safety, as set out by the World Health Assembly (Resolution 76.17 on the impact of chemicals, waste, and pollution on human health) and the European Environment and Health Process (EHP) [30]. The EHP Partnership on HBM, or HBM Partnership, launched at the Seventh Ministerial Conference on Environment and Health in 2023, currently involves over 20 countries, including Ireland [31].

Ireland still lacks a national HBM programme, placing it behind EU counterparts such as Germany, Belgium, and France. Irish researchers have conducted targeted pilot studies—including mercury detection in maternal hair [32], polychlorinated dioxins and furans (which have shown a declining trend since the 2008 dioxin incident in Ireland) [33], flame retardants in breast milk [34], as well as glyphosate exposures among amenity horticulturists [35,36] and the general population [37,38]. Although, these efforts remain isolated and fragmented, lacking integration into a cohesive national biomonitoring strategy. For example, a 2014 cohort study revealed that 79% of mothers had detectable mercury levels (mean: 0.262 µg/g), with fish consumption and socioeconomic status being key correlates [39]. This highlights the importance of localised studies but also highlights the absence of a unified framework to harmonise data collection, share insights, and inform broader public health policies. Similarly, phthalate metabolites were found ubiquitously in participants of the DEMOCOPHES (Demonstration of a Study to Coordinate and Perform Human Biomonitoring on a European Scale) pilot study, with higher concentrations associated with PVC exposure and fast-food consumption [32]. These findings demonstrate Ireland’s capacity to carry out effective HBM research but also emphasise the pressing need for the establishment of a comprehensive, long-term national HBM programme.

To achieve these ambitions, Ireland has taken the first steps toward developing a national human biomonitoring programme by conducting a feasibility study [40]. The HBM4IRE (Human Biomonitoring for Ireland) project, funded by Ireland’s Environmental Protection Agency (EPA), aims to bridge this gap by establishing criteria for a national human biomonitoring framework. A critical first step is prioritising chemicals of concern, a complex task requiring alignment with EU priorities (e.g., HBM4EU’s 18 substance groups and the PARC priority list) while also addressing Ireland’s unique exposure landscape. Globally, nations balance scientific, regulatory, and societal factors in chemical prioritisation. For instance, Belgium prioritised chemicals like POPs (Persistent Organic Pollutants) and PAHs (Polycyclic Aromatic Hydrocarbons) for their HBM programme due to widespread exposure and potential harm, with a focus on vulnerable populations and existing policies to guide targeted interventions [41]. Slovenia targets pollutants in industrial “hotspots” [42], France employs Delphi consensus methods to integrate public concern [43], and the U.S. National Health and Nutrition Examination Survey (NHANES) programme prioritises high-risk agents like lead. HBM4EU further demonstrates the value of multi-stakeholder engagement, combining hazard data, policy relevance, and biomarker feasibility to rank and prioritise chemicals of concern [23]. In addition, Latvia’s HBM4LV programme used a six-step process for chemical prioritisation, reviewing 318 chemicals and shortlisting 130 based on health impact, hazardous properties, and national relevance. The adapted Hanlon method identified 30 high-priority substances, focusing on pesticides, heavy metals, and persistent organic pollutants [44]. These prioritisation methods aim to address both historical and emerging chemical concerns while considering logistical constraints [43,45].

It is important to note that the public’s concern about certain chemicals significantly influences their selection in HBM programmes across various countries. When there is substantial public demand for monitoring specific substances, HBM programmes often respond by including these chemicals in their studies to address societal concerns. Incorporating citizen viewpoints and insights is a key element of a systematic, transparent, and participatory strategy within the Human Biomonitoring Initiative for Europe [46,47].

This study presents Ireland’s first systematic chemical prioritisation process for a HBM programme, harmonising European frameworks with public perception about chemicals of concern. We propose a dual approach: (1) leveraging the HBM4EU/PARC priority list to identify EU-wide priority chemicals and (2) incorporating Irish public insights through a national survey. Through the HBM4IRE chemical prioritisation study, Ireland can establish a scientifically robust and contextually relevant chemical priority list, setting the foundation for a national HBM programme, which can also be adapted as guidance for other EU and non-EU countries based on their unique needs.

## 2. Materials and Methods

**Figure 1:**
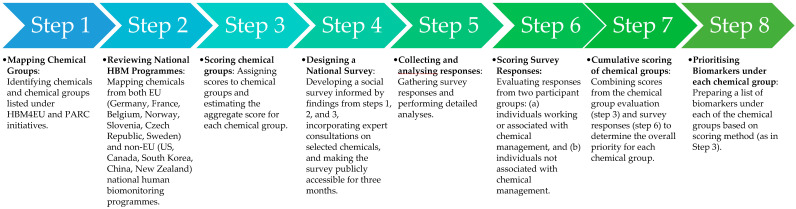
Steps followed in chemical prioritisation under the HBM4IRE project.

The chemical prioritisation methodology for Ireland’s national HBM programme involved a multi-step approach designed to identify and prioritise chemicals based on both international best practices and the perception of people living in Ireland. This process combined a review of chemicals listed in the existing HBM programmes with structured scoring. The stepwise prioritization process adopted in the study is presented in Figure 1.

To initiate the chemical prioritisation process, we first incorporated chemicals with European-wide concerns and ensured alignment with high-priority substances recognised at the EU level. For this, we mapped chemical groups from two significant European initiatives, the European Joint Programme HBM4EU and the PARC initiative, by cross-referencing chemicals identified as priority chemicals in these projects. In the next step, we conducted an extensive review of HBM programmes from multiple countries, including EU countries (Germany, France, Belgium, Norway, Slovenia, Czech Republic, and Sweden) and non-EU (US, Canada, South Korea, China, and New Zealand) national human biomonitoring programmes, identifying their prioritised chemicals (analysed in their most recent cycle of HBM programme) to gain insight into global trends and emerging chemical concerns.

Once an initial list of chemical groups was established, a scoring system was applied to objectively rank them in Equation (1). Chemicals frequently prioritised in national and international HBM programmes were assigned higher cumulative scores. Specifically, chemicals listed under both the HBM4EU and PARC were assigned a score of 2, while those prioritised in either initiative received a score of 1. Chemicals prioritised in HBM programmes of other EU countries were assigned a score of 1, whereas those from non-EU countries received a score of 0.5. This scoring system was developed to rank chemical groups based on their frequency of prioritization.

$$s_{j,c}=\left\{\begin{array}{c} 2\;if\;prioritized\;in\;both\;HBM4EU\;and\;PARC\;list\;of\;priority\;chemicals\\ \;1\;if\;prioitized\;in\;HBM4EU\;or\;PARC,\;or\;EU\;national\;HBM\;programs\\ 0.5\;if\;prioritized\;in\;non-EU\;national\;HBM\;programs\;globally \end{array}\right.$$

where $s_{j,c}$ is the score of a chemical “j” for the country “c”, where c ϵ [list considered EU countries (Germany, France, Belgium, Norway, Slovenia, Czech Republic, and Sweden) and non-EU (US, Canada, South Korea, China, and New Zealand) countries].

The cumulative prioritisation score (C_j_) for each chemical of concern identified in the study is as follows:

(1) $$C_{j}=\sum_{c=1}^{n}s_{j,c}$$

where j ϵ [list of chemicals identified], and n = total number of countries considered in the study.

With a preliminary list of chemical groups identified, we developed a national survey tailored to the Irish context (a snippet of the survey can be found in Supplementary Materials S1). Building on the HBM4EU social survey, we adapted the questionnaire to assess the public awareness and perception of various chemical groups, including five heavy metals. The national HBM4IRE survey was designed using SurveyMonkey (SurveyMonkey is a widely used online survey platform that allows users to design, distribute, and collect responses for surveys) and circulated via email and online posts on institutional homepages and social media accounts of University College Dublin and EPA Ireland (from July to September). This was aimed at evaluating participants’ level of awareness and understanding of chemicals, providing insights into their perceptions of potential harmfulness. Participant recruitment followed a random sampling approach. Eligible participants were adult citizens living in Ireland (18 years or older). Participants were provided with information on the HBM4IRE project, the survey’s purpose, and consent requirement within the online survey platform.

To ensure comprehensive data collection, the survey was designed to capture the perspectives of both individuals working in or associated with chemical management and members of the general public with no direct involvement in the field. An aggregate score for each chemical was subsequently estimated based on respondents’ perception of its harmfulness. The Perception of Harmfulness was quantified using a scaled approach, where higher values corresponded to greater perceived harmfulness. Table 1 outlines the scale used to categorise levels of perceived harmfulness:

Thereafter, corresponding to responses for each chemical, an aggregate perceived level of harmfulness was estimated as per Equation (2):

(2) $$\{NS_{j}\}=\sum_{i=1}^{5}p_{i}*\;hs_{i}$$

where $NS_{j}$ is the cumulative national score for chemical j; “$p_{i}”$ is the proportion of people who responded for a perceived level of harmfulness {${\mathrm{hs}}_{i}$}; and i ϵ [1, 5].

Notably, survey responses were categorised into experts (individuals working in or associated with chemical management) and non-experts (individuals with no direct involvement in chemical management) based on a self-reported response to a survey question. To account for differences in knowledge and familiarity with chemicals, we applied a weighted scoring system. A weight of 0.7 (70%) was assigned to expert responses. In contrast, non-expert responses were assigned a weight of 0.3 (30%), reflecting their relatively lower familiarity with chemical risks. We conducted a sensitivity analysis by varying the non-expert weightage and recalculating chemical scores, confirming the robustness of our framework. The details are in Supplementary Materials S2. The aggregate survey score for chemicals was derived using Equation (3):

(3) $$W_{j}=w_{e}\times H_{e,j}+w_{ne}\times H_{ne,j}$$

Noteworthily, separate scores were calculated for experts ($H_{e,j}$) and non-experts ($H_{ne,j}$) using the approach mentioned in general for {$NS_{j}$}. ($H_{e,j}$) is the cumulative score for chemical “j” estimated for the expert group using Equation (2). Likewise, ($H_{ne,j}$) corresponds to the score for non-experts. Thereafter, a weighted composite score ($W_{j}$) was estimated.

The next step involved synthesising the survey results by estimating the overall score for each chemical group and compiling a comprehensive list to identify the top 10 priority chemical families for Ireland’s national HBM programme. This shortlist integrates the proportion of chemicals selected by the public, ensuring that the biomonitoring programme addresses both societal concerns and chemicals identified as priorities based on public perception.

The final step involved combining the scores derived from the social survey with those assigned to chemicals in HBM4IRE, PARC, and country-specific national HBM programmes. By summing these scores, we estimated the final aggregate score for each chemical group, providing a data-driven basis for prioritisation. Final aggregate scores were calculated by using Equation (4):

(4) $$F_{j}=C_{j}+W_{j}$$

where (F_j_) represents the final aggregate score, (C_j_) represents EU prioritisation scores, and (W_j_) represents weighted perception scores estimated from the national survey.

Once the list of chemical groups was established, the next objective was to identify priority biomarkers within each group for Ireland’s national HBM programme. To achieve this, we adopted the same approach used for chemical prioritisation, reviewing biomarkers listed in HBM4IRE, PARC, and other national HBM programmes. After consolidating an initial list of biomarkers within each chemical group, we applied a scoring system to objectively rank them. Biomarkers frequently prioritised in national and international HBM programmes were assigned higher cumulative scores.

$$b_{j,k,c}\;=\left\{\begin{array}{c} 2\;if\;listed\;in\;both\;HBM4EU\;and\;PARC\;priority\;biomakers\;list\\ 1\;if\;listed\;in\;HBM4EU\;or,\;PARC\;or,\;EU\;national\;programs\\ 0.5\;if\;listed\;in\;non-EU\;national\;programs\;biomarker\;list \end{array}\right.$$

where $b_{j,k,c}$ represents the score for each biomarker “k” identified under chemical “j” for country “c”.

Thereafter, the cumulative score (B_j,k_) for each biomarker identified for each chemical of concern is estimated as shown in Equation (5):

(5) $$B_{j,k}=\sum_{c=1}^{n}b_{j,k,c}$$

where j ϵ [list of chemicals identified], k ϵ [list of biomarkers identified under each chemical “j”], and n = total number of countries considered in the study.

## 3. Results

### 3.1. National Survey: Demographics

The survey captured 218 participants, predominantly urban (62%) and long-term residents (82% in Ireland >10 years), ensuring the representation of generational exposure perspectives. Participants were highly educated (89% holding a bachelor’s or advanced degrees) and professionally diverse: 55% worked in government roles, 17% in the private sector, and 44% in scientific or technical fields, with 36% directly involved in chemical management (e.g., regulatory, laboratory roles). While 20% of respondents expressed extreme concern about chemical exposure, 43% reported moderate concern, suggesting risk perception is influenced by occupational familiarity. This demographic profile, marked by higher education, professional engagement, and urban residency, highlights the credibility of responses but also highlights disparities in public awareness, particularly those outside scientific sectors.

### 3.2. Prioritisation of Chemical Groups in the International HBM Programmes

A comprehensive review of existing human biomonitoring (HBM) initiatives from multiple countries was initially conducted to establish the priority chemical groups for Ireland’s national HBM programme. The studies and internet sources reviewed for each country are presented in Supplementary Materials S3. The cumulative scores assigned to each chemical group were derived from the number of international and national programmes that included them as priority chemicals. The analysis identified lead, cadmium, and plasticisers as the most frequently prioritised chemical groups, each receiving the highest cumulative score of 11.5, consistent with the HBM review. Other high-priority chemicals, such as mercury (10.0), bisphenols (10.5), and pesticides (8.5), also demonstrated widespread recognition as chemicals of concern. Additionally, flame retardants and perfluoroalkyl and polyfluoroalkyl substances (PFASs) emerged as priority chemicals with a cumulative score of 9.0.

Several chemical groups were identified as moderate priorities based on their inclusion in a smaller number of human biomonitoring programmes. Polycyclic aromatic hydrocarbons (PAHs) (7.0), arsenic (7.5), chromium (6.0), and persistent organic pollutants (POPs) (5.0) were among the chemicals with lower but notable cumulative scores. Other groups, including volatile organic compounds (VOCs), UV filters, solvents, parabens, and tobacco alkaloids, were also recognised. However, they received comparatively lower scores due to their variable prioritisation across different national and international programmes.

**Figure 2:**
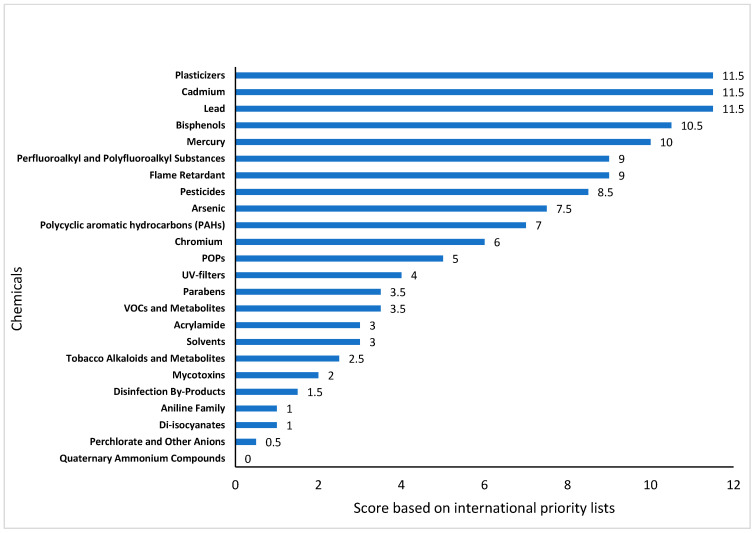
Scoring of chemicals based on the HBM4EU, PARC, and national HBM programmes.

A few chemical groups exhibited limited prioritisation in biomonitoring programmes worldwide. Acrylamide, mycotoxins, disinfection by-products, di-isocyanates, and the aniline family received cumulative scores equal to or below 3.0 (Figure 2).

### 3.3. Chemical Prioritisation Based on the National Survey

A total of 218 people participated in the HBM4IRE national survey. Of these, 185 participants (85% completion rate) fully completed the survey and were included in the final analysis. The respondents were categorised based on their professional affiliation. A total of 63 participants (34%) reported that they were associated/working in chemical management, including regulatory, industrial, or research roles. In contrast, 122 participants (66%) had no association with chemical management.

**Figure 3:**
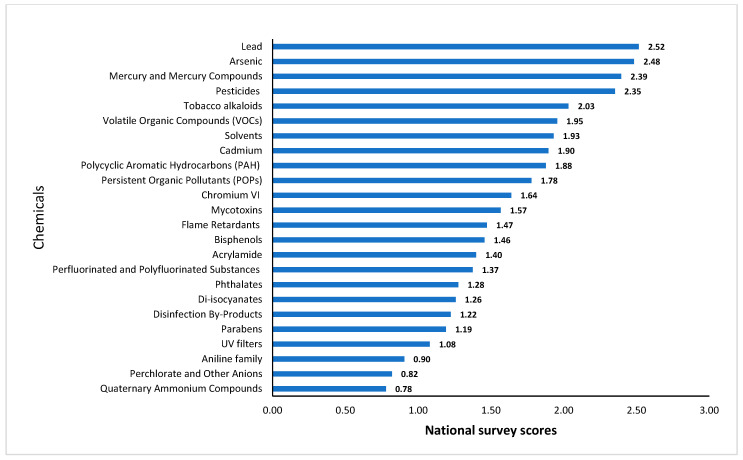
Scoring of chemicals based on the national survey among the Irish population.

The national survey highlighted key chemicals of concern based on public perception. Heavy metals such as lead (2.52), arsenic (2.48), and mercury (2.39) ranked highest, indicating a strong public awareness of their toxicity. Pesticides (2.35) and tobacco alkaloids (2.03) also scored highly, emphasising concerns about exposure from food and lifestyle sources (Figure 3).

VOCs (1.95), solvents (1.93), and persistent organic pollutants (1.78) were perceived as moderately hazardous, reflecting awareness of industrial and environmental pollutants. Emerging contaminants such as bisphenols (1.46), flame retardants (1.47), and PFASs (1.37) received moderate concern. Lower-ranked chemicals, including quaternary ammonium compounds (0.78) and perchlorate (0.82), suggested limited public awareness. These results were integrated into the prioritisation process to ensure Ireland’s human biomonitoring programme reflects both scientific and societal concerns.

### 3.4. Aggregate Scoring of Chemical Groups

**Figure 4:**
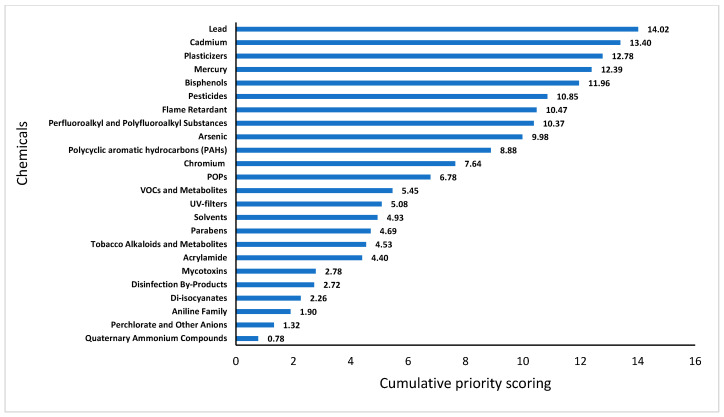
Final scoring of prioritised chemicals/chemical groups based on cumulative scores.

After integrating the national survey and global HBM programmes/initiatives, aggregate scores and the final prioritisation were conducted (Figure 4). Lead (14.02) emerged as the top priority chemical group, with significant concern across various regions, including strong public support in the social survey. This was followed closely by cadmium (13.40), which also received substantial weight from public and expert inputs. Similarly, other hazardous chemical, such as plasticisers (12.78), mercury (12.39), and bisphenols (11.96), ranked highly, indicating widespread concern over their impact on public health.

Emerging contaminants, such as perfluoroalkyl and polyfluoroalkyl substances (PFASs) (10.37) and flame retardants (10.47), were also prioritised, reflecting the growing awareness of these chemicals in consumer products and environmental exposure. Pesticides (10.85) and arsenic (9.98) were similarly high in the final ranking, reflecting their relevance in food safety and environmental contamination. The lowest-ranking chemicals, such as quaternary ammonium compounds (0.78) and perchlorate (1.32), were prioritised to a lesser extent, indicating their relatively lower perceived risks in the context of human biomonitoring.

### 3.5. Selection of Biomarkers Under Each Priority Chemical Group

**Figure 5:**
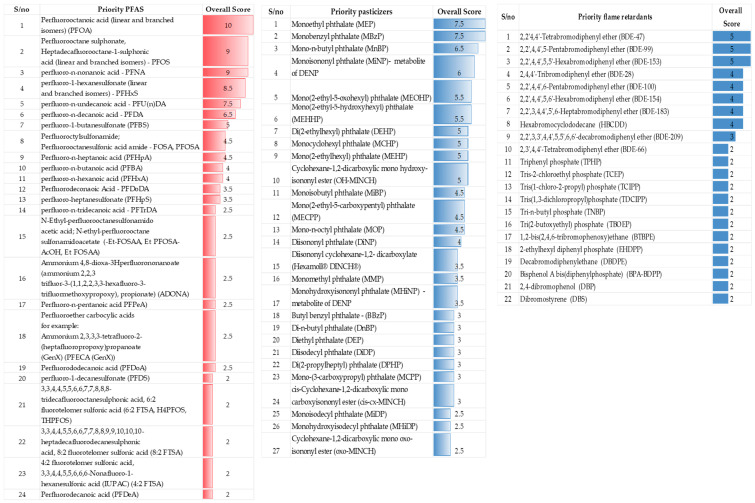

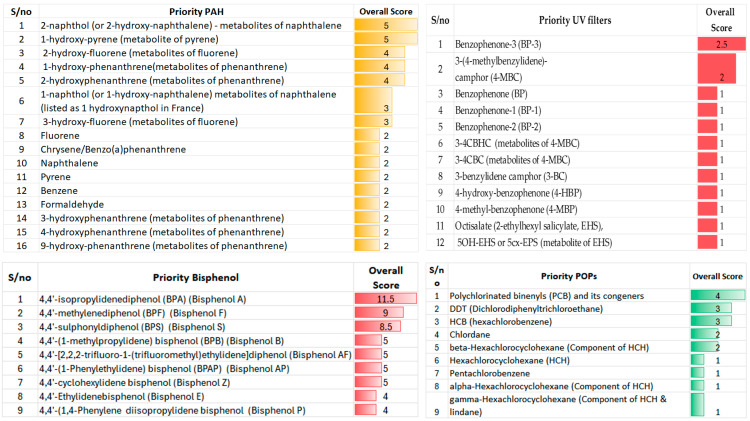

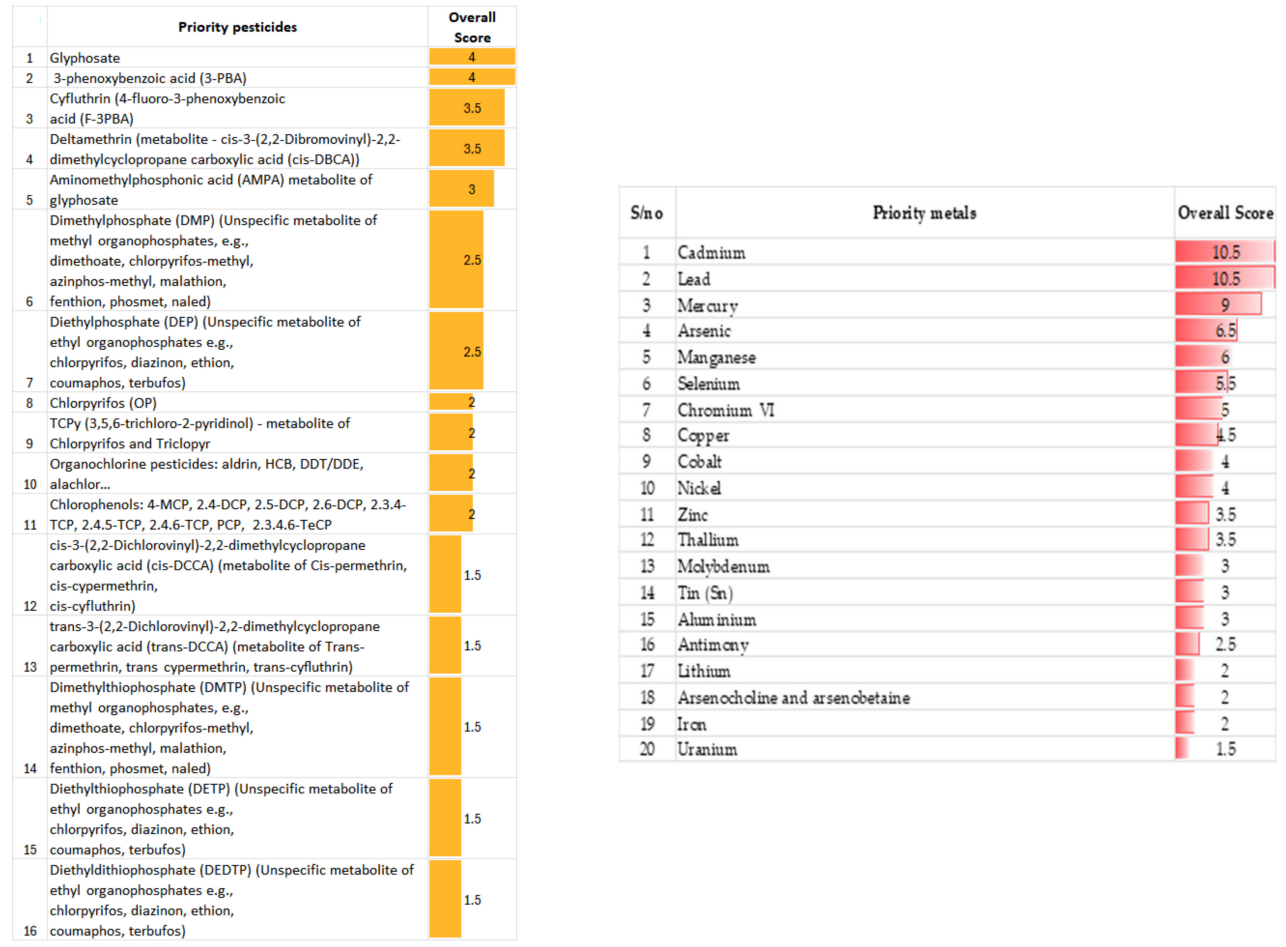
List of priority biomarkers of major chemical groups.

The priority biomarkers identified based on their inclusion in key HBM programmes and initiatives are systematically presented in Figure 5. The biomarker list organises the chemicals based on their priority level, derived from the cumulative scoring based on their appearance in the reviewed HBM programmes (excluding Norway—which was excluded from the biomarkers prioritisation process due to a lack of information in the public domain).

For example, in the case of bisphenols, seven priority chemicals/biomarkers were selected, with Bisphenol A (BPA) ranking first with an overall score of 11.5. Other bisphenols of priority included Bisphenol F (BPF), Bisphenol S (BPS), and Bisphenol B (BPB), with decreasing scores based on their environmental persistence, exposure levels, and associated health concerns. The complete list includes a range of bisphenols, such as Bisphenol AF, Bisphenol AP, and Bisphenol Z, all of which are widely used in various industrial applications but with varying degrees of concern regarding their toxicity and biomonitoring feasibility. Similarly, the biomarkers of other chemical groups were selected according to their overall scores, which reflect their relevance in ongoing HBM programmes across the globe, helping to focus monitoring efforts on substances that have the most significant implications for human health. The detailed figure in the study provides a comprehensive list of these chemicals, along with their corresponding priority rankings.

Also, Benzene, along with its urinary metabolites N-Acetyl-S-(phenyl)-L-cysteine (S-PMA) and trans, trans-Muconic acid (tt-MA), are commonly analysed in HBM programmes, among other biomarkers of VOCs.

## 4. Discussion

The World Health Organization’s (WHO) ambitions to promote nations developing national human biomonitoring (HBM) programmes as a tool to evaluate chemical pollution, inform citizens transparently, and promote safety require a chemical prioritisation process [28]. Major advancements in HBM strategies have occurred in Europe, in particular, due to EU-wide initiatives such as the HBM4EU and PARC, which also include a thorough chemical prioritisation process [48]. To incrementally build on these advancements rather than repeating these processes, each EU country should align with the EU priorities while still taking account of their respective chemical priorities. This was a core objective of the HBM4IRE project, and a chemical prioritisation was conducted by estimating the aggregate scores derived from three key sources: (1) substances prioritised under EU frameworks (the HBM4EU and PARC), (2) chemicals identified in national and international HBM programmes, and (3) public perception data from a national Irish survey. Notably, this study did not conduct independent hazard assessments but instead leveraged pre-existing prioritisation efforts embedded within EU initiatives. Our prioritisation process was based on the chemicals already prioritised through rigorous, peer-reviewed processes (in the HBM4EU), including hazard characterisation, exposure analysis, and policy relevance assessments [23]. The HBM4IRE chemical priority methodology ensures alignment with evidence-based regulatory frameworks while avoiding redundancy.

The initial steps of this process involved reviewing EU-wide initiatives (i.e., the HBM4EU and PARC), followed by a review of established national HBM programmes. The study identified 12 national HBM programmes and it showed a global consensus on high-risk chemicals driven by their persistence, toxicity, and regulatory relevance. Lead, cadmium, and plasticisers emerged as top priorities as they were consistently included in EU-specific initiatives (the HBM4EU and PARC) and featured prominently in several national HBM programmes, including those in Sweden, Germany, France, Belgium, and the Czech Republic. These chemicals are monitored worldwide due to their well-documented health impacts, including neurodevelopmental deficits, renal toxicity, and endocrine disruption [49,50,51]. Mercury followed closely, reflecting alignment with the Minamata Convention [52] and heightened monitoring in regions with seafood-dependent diets, such as Sweden and Germany [53]. European HBM programmes also prioritise bisphenols and pesticides, highlighting regulatory action on endocrine disruptors and agrochemical risks [45,54]. PFASs are also prioritised under the HBM4EU initiative and EU countries along with many HBM programmes in non-EU nations like the US, Canada, China, and South Korea due to their high exposure prevalence and health risks, urging regulatory action to limit exposure. In the U.S., the Environmental Protection Agency (EPA) has developed an extensive PFAS Action Plan to address these chemicals, focusing on regulatory actions, research, risk communication, and collaboration with state, local, and tribal governments [55]. There are also emerging chemicals of concern, such as perchlorates and quaternary ammonium compounds (QACs), that, to date, have received minimal attention, though research shows that emerging contaminants like disinfectant-derived QACs have been potentially linked with antimicrobial resistance. These results demonstrate that countries need to identify localised risks, such as legacy heavy metals in aging infrastructure and agricultural pesticides, while also reviewing these chemical priorities at regular intervals to ensure the inclusion of novel and emerging chemicals.

Importantly, the lower prioritisation of certain chemicals in HBM programmes could be a reflection of being novel or emerging contaminants, which highlights the necessity for regular HBM cycles. Each HBM cycle would necessitate a chemical review so that the programme captures up-to-date data on novel and emerging chemicals of concern. This proactive approach is vital to track substances that may enter human populations as industrial, agricultural, or consumer practices evolve.

Integrating expert and public inputs enhances the acceptability and effectiveness of chemical management strategies [56,57] by engaging all stakeholders in chemical safety, particularly the public, to identify societal needs, guide research priorities, ensure transparency, and enhance societal benefits in HBM [24,47]. Previous studies have shown that citizens are highly concerned about chemical exposures, particularly through food, water, and air [46], and previous citizen surveys have shown strong public support for HBM as a tool to assess chemical exposure and inform health policies [58] with the majority of respondents viewing HBM as reliable (84%) and necessary (81.7%), with a need for increased coordination at European (86.2%) and national levels (83.7%). Citizen perspectives are integral into the systematic and participatory approach of HBM initiatives such as the HBM4EU. The HBM4IRE national survey was adapted from the HBM4EU survey [59]. The survey achieved a high completion rate of 85%, indicating strong participant engagement. However, 15% of respondents did not complete the survey, which may be attributed to factors such as the length and complexity of the questionnaire. Longer surveys can lead to respondent fatigue, reducing the likelihood of full participation, particularly if questions require detailed responses or technical knowledge, as reported in many studies [60,61]. The survey’s demographic information revealed a well-educated, professionally diverse participant group, with approximately a third involved with chemical management, which increases the credibility of the survey findings. The respondents who indicated working in chemical management were more highly weighted, as professional engagement in chemical management correlates with higher awareness of chemical hazards [62], as they often base risk evaluations on empirical data, though their assessments may diverge from public perceptions [63]. However, it is still important to capture public concerns and societal perceptions of harm [64].

The national survey revealed that public concern aligned with well-established hazards, such as heavy metals (lead, arsenic, and mercury), due to regulatory measures like leaded petrol bans [64]. Additionally, pesticides were of public concern, which reflects Ireland’s agricultural industry. Though farmers typically recognize the harmful effects of pesticides on health and the environment, this knowledge often does not translate into safe practices [65], and there have been Irish studies that identified pesticide exposures among workers [35,36] and the general public [37,38]. Increased public concern may also arise from debates on EU pesticide policies, such as the Farm-to-Fork Strategy’s target to halve chemical pesticide use by 2030 [66]. VOCs and tobacco alkaloids are also highly prioritised by the Irish public, likely due to urban traffic emissions and public health campaigns on smoking. The survey also highlighted gaps in awareness of emerging contaminants like UV filters, phthalates, and PFASs. Alongside measurements, there is also a need for regional HBM campaigns and updated risk communication to disseminate information on exposures and mitigation successes for legacy and emerging chemicals.

Finalising a chemical priority list involved the integration of global HBM programme priorities with Ireland’s national survey data under the HBM4IRE project, revealing many alignments such as lead, cadmium, and plasticisers emerging as top priorities. The discrepancies that were identified were POPs and chromium, which are extensively monitored in global HBM programmes [67,68,69,70,71] due to their environmental persistence and carcinogenicity. However, these concerns were not reflected in public responses, highlighting a disconnect between scientific urgency and public awareness. Conversely, arsenic ranked highly in the national survey despite limited inclusion in global HBM programmes, reflecting community-driven concerns in regions with localised contamination, such as areas impacted by historical mining or naturally occurring groundwater arsenic. For instance, arsenic contamination reported in Irish groundwater has emerged as a significant concern, with some areas exceeding the WHO limit of 10 μg/L [72]. The occurrence of arsenic is strongly linked to poorly productive aquifers, and fractured bedrock aquifers also show elevated arsenic levels [73]. The prioritisation process revealed unrecognised risks, such as quaternary ammonium compounds (QACs), which scored low despite increased disinfectant use during the COVID-19 pandemic [74,75] and links to antimicrobial resistance [76,77]. Similarly, PFASs, which are prioritised globally under HBM programmes for their persistence, remain under-recognised in public discourse in Ireland, highlighting the need for proactive communication strategies.

It is important to note that the prioritisation process adopted in this study is focused on substances for which established, sensitive, and specific biomarkers are already available, ensuring feasibility for immediate integration into a national biomonitoring programme. This pragmatic approach aligns with methodologies employed in other international frameworks, such as the German prioritisation strategy, where substances of toxicological concern are first identified, followed by the targeted development of analytical methods for cases where existing biomarkers are inadequate [78]. For instance, DINCH (Diisononyl cyclohexane-1,2-dicarboxylate) metabolites, emerging substitutes for traditional phthalates, required novel method development to enable accurate biomonitoring, as demonstrated in the German approach. While the current Irish priority list emphasises chemicals with validated biomarkers (e.g., heavy metals, phthalates, and PFASs), the inclusion of emerging substances like QACs or UV filters may necessitate similar innovation in future phases. This dual focus, leveraging existing markers for immediate implementation while acknowledging the need for method development for understudied chemicals, ensures adaptability to evolving exposure risks. The framework mirrors the German model’s emphasis on balancing scientific relevance with technical feasibility, ensuring human biomonitoring programmes remain responsive to both legacy contaminants and newly identified threats.

The study produced a final list of priorities for the national HBM programme: heavy metals (lead, cadmium, mercury, arsenic, chromium VI), plasticisers (phthalates), bisphenols, pesticides, flame retardants, perfluoroalkyl and polyfluoroalkyl substances (PFASs), polycyclic aromatic hydrocarbons (PAHs), persistent organic pollutants (POPs), volatile organic compounds (VOCs) and UV filters. The chemicals shortlisted included legacy chemicals which are persistent in the environment (i.e., flame retardants and some pesticides), to banned substances (i.e., POPs), and more emerging issues (i.e., UV filters). Human biomonitoring is the only method that can identify accumulative exposures to exposures that occur from multiple sources, such as consumer products (i.e., plasticisers, bisphenols, PFASs, and UV filters) or foodstuffs (i.e., pesticides and plasticisers). The study grouped heavy metals, as these can typically be analysed using multi-analysis methods. Other chemicals and their biomarkers were grouped separately. While this is the first iteration of the list, it should be regularly updated to reflect new research, changing trends in chemical exposure, and emerging risks. The establishment of this prioritised list serves as an essential foundation for Ireland’s HBM programme, which aims to monitor and assess chemical exposure levels in the population and guide informed policy decisions aimed at mitigating these risks.

The importance of selecting appropriate biomarkers to reflect exposures has been previously highlighted [79]. Several biomarkers within each chemical group are prioritised in national HBM programmes, and in the current study, it is essential to highlight that multiple biomarkers within a chemical group can often be analysed simultaneously. This approach, referred to as multi-analysis methods, allows for the simultaneous assessment of multiple biomarkers from a single biological sample. This not only facilitates the comprehensive evaluation of biomarkers within a chemical group but also significantly reduces analytical costs, making it a cost-effective solution for large-scale HBM programmes. For example, for phthalates, a UHPLC-MS/MS method was developed for analysing 23 metabolites, including 20 phthalate and 3 DINCH metabolites [80].

Legacy chemicals, such as POPs, continue to present challenges due to their historical usage, persistence in the environment, and bio-accumulative nature and are included in the priority chemical lists of HBM programmes of countries like the Czech Republic, Belgium, and Sweden. Although banned or restricted under international conventions such as the Stockholm Convention, these substances remain pervasive in ecosystems due to their resistance to degradation. Their inclusion in HBM programmes is essential for understanding long-term exposure risks and monitoring the enforcement of existing regulations and remediation efforts. Even with chemicals that may be restricted or banned, it is important to evaluate their lasting effects and to ensure no reoccurrence of exposures. Recent research by Kasper-Sonnenberg et al. (2025) reported a significant decline in exposure to legacy phthalates such as DnBP and DEHP in Germany over 35 years (1988–2022), with reductions exceeding 90%. However, rising exposure to substitutes like DINCH and DEHTP reflects the evolving landscape of chemical risk and time trends [81].

Similarly, emerging chemicals, including UV filters and their derivatives, have been listed in the HBM4EU and Germany, Sweden, and South Korea priority lists due to their widespread use and potential health impacts. Common UV filters analysed in HBM programmes are Benzophenone-3 (BP-3), also known as oxybenzone, and 3-(4-methylbenzylidene)-camphor (4-MBC), both of which are widely used in sunscreens and personal care products to protect against harmful UV radiation [82,83,84]. Benzophenone (BP) and its derivatives, including BP-1 and BP-2, are also prevalent in cosmetics, plastics, and packaging for their UV-absorbing properties [85]. Consequently, these chemicals have been prioritised in initiatives like HBM4IRE, PARC, and European HBM programmes.

These findings highlight the critical role of HBM in monitoring both legacy and emerging chemicals, as demonstrated by initiatives like the HBM4IRE, PARC, and other European HBM programmes to enable comprehensive exposure assessment and effective risk management.

This study provides valuable insights into chemical prioritisation for Ireland’s HBM programme, but a few limitations must be acknowledged. One key limitation is the sample size of the social survey, which included approximately 218 participants. Given Ireland’s total population, this sample may not be fully representative of national perception patterns and public awareness about chemicals of concern. Also, future surveys could consider strategies such as questionnaire simplification, adaptive questioning (where respondents only see relevant questions), or breaking up the survey into shorter sections to further improve completion rates. Expanding future surveys to include a more diverse and nationally representative participant pool with targeted outreach would enhance the robustness of assessments and may help ensure a more balanced representation of people.

It is important to note that the chemical priority lists established within this study were explicitly evaluated for HBM programmes. Not all substances/chemicals can be analysed using HBM analytical methods; thus, there may be chemicals of national concern not included in the national survey design and the chemical list, as this study is focused on chemicals that HBM can analyse. With rapid advancement in the development of HBM analytical methods, regular reviews may capture these chemicals. However, it is vital to develop an initial priority list prior to initiating a national HBM programme, and building on international expertise can provide the best overview.

This study mapped priority lists throughout the world and relied on robust reviews for evaluating priority chemicals. Given the rapid advancement in chemical applications and the emergence of new contaminants and their substitutes, it is crucial to regularly update chemical prioritisation lists to effectively address evolving environmental and health risks. Long-term ambitions and goals include establishing a sustainable and structured HBM programme with periodic assessments, which will enable continuous monitoring of chemical exposures. Integrating and investing in advanced analytical techniques, such as high-throughput screening and non-targeted analysis, will further enhance the detection of emerging chemicals of concern.

Future research should also consider the socioeconomic impacts of chemical exposures, particularly among vulnerable populations. Strengthening collaborations between regulatory agencies, research institutions and institutes, and public health bodies can ensure that Ireland’s HBM programme remains adaptive and responsive to evolving environmental and public health challenges. Continuous surveillance and periodic reassessments will be key to capturing emerging contaminants and informing evidence-based policy decisions.

## 5. Conclusions

These research findings mapped priority lists across EU initiatives (i.e., the HBM4EU and PARC) for national HBM programmes worldwide to develop an initial list of priority chemicals for an Irish national HBM programme. This approach could be utilised by each country seeking to initiate or reevaluate their chemical priority lists for HBM programmes.

In addition, the varying levels of concern about chemical exposure indicated the need for tailored awareness initiatives to address public perceptions and promote understanding of chemical safety. This demographic foundation provides a critical context for interpreting the survey results and shaping future strategies in chemical monitoring and management.

Bridging this gap requires targeted efforts by Ireland’s HBM programme, including educational campaigns aimed at raising awareness about the persistence and health impacts of legacy chemicals, such as POPs, and the risks associated with emerging substances, like UV filters. Collaborative efforts with international HBM networks can further align priorities and promote shared best practices for addressing these challenges.

## Figures and Tables

**Table 1:** Score scale used to categorise levels of perceived harmfulness.

Perception of Harmfulness	Seriously Harmful	Moderately Harmful	Slightly Harmful	Not Harmful at All	Don’t Know
Scale	3	2	1	0	0

## Data Availability

The original contributions presented in this study are included in the article/Supplementary Materials. Further inquiries can be directed to the corresponding author.

## Supplementary Materials

The following supporting information can be downloaded at: https://www.mdpi.com/article/10.3390/toxics13040281/s1, Supplementary Material S1: Snippets of National Survey. Supplementary Material S2: Sensitivity Analysis. Supplementary Material S3: HBM Programme Review. References [23,42,45,86,87,88,89,90,91,92,93,94,95,96,97,98,99,100,101,102,103,104,105,106,107,108,109,110] are cited in the Supplementary Material.

## Abbreviations

The following abbreviations are used in this manuscript:

HBM	Human Biomonitoring
HBM4IRE	Human Biomonitoring for Ireland
HBM4EU	Human Biomonitoring for Europe
PARC	Partnership for the Assessment of Risks from Chemicals
EEA	European Environment Agency
WHO	World Health Organisation
PAH	Polycyclic Aromatic Hydrocarbons
POPs	Persistent Organic Compounds
DINCH	Di (isononyl) cyclohexane-1,2-dicarboxylate
PFASs	Per- and Polyfluoroalkyl Substances
NHANES	National Health and Nutrition Examination Survey
CDC	Centres for Disease Control and Prevention
GerES	German Environmental Survey
DEMOCOPHES	Demonstration of a study to coordinate and perform human biomonitoring on a European scale
