# Supplementary material for: Chemical Prioritisation for Human Biomonitoring in Ireland: A Synergy of Global Frameworks and Local Perspectives
Source: Toxics. 2025 Apr 7;13(4):281. doi: 10.3390/toxics13040281 (PMC12031266; doi:10.3390/toxics13040281)
Supplement: Supplementary file 1 [file toxics-13-00281-s001.zip › toxics-3551292-supplementary.pdf]

Supplementary Material

Type of the Paper: Article

Chemical Prioritisation for Human Biomonitoring in Ireland: A Synergy of Global Frameworks and Local Perspectives.

Richa Singh [1], Holger. Martin. Koch [2], Marike. Kolossa-Gehring [3] and Alison Connolly [1,*]

[1] UCD Centre for Safety & Health at Work, School of Public Health, Physiotherapy and Sports Science, University College Dublin, D04 V1W8, Dublin, Ireland
[2] Institute for Prevention and Occupational Medicine of the German Social Accident Insurance, Institute of the Ruhr-University Bochum (IPA), Bürkle-de-la-Camp-Platz 1, 44789 Bochum, Germany
[3] German Environment Agency (Umweltbundesamt), 06844 Dessau-Roßlau, Germany

* Correspondence: alison.connolly@ucd.ie; Tel.: (+353 17163456)

Supplementary Material S1: Snippets of National Survey

HBM4IRE epaResearch UCD DUBLIN

Knowledge and Perception of Chemical Substances

Please rate the following substances based on your knowledge of them.

* 15. Do you Know - Phthalates (Commonly used as plasticizers in the manufacturing of flexible plastics, such as in PVC products, flooring, and wall coverings.)

○ Yes

○ No

16. If yes, could you select one of the options below on how you perceive this substance to be harmful to people?

	Seriously harmful	Moderately harmful	Slightly harmful	Not harmful at all	Don't know
Phthalates	○	○	○	○	○

Supplementary Material S2: Sensitivity Analysis

To thoroughly assess the sensitivity of our results, we conducted a systematic analysis by adjusting the weight assigned to non-expert responses across a broad range (5% to 50%) and recalculating the overall scores for each chemical. This exercise revealed that the prioritisation framework is largely robust to variations in weightage given to the non-expert contribution.

For the majority of chemicals (>80%), the dispersion of scores around the mean remained minimal (e.g., <5% deviation), indicating low sensitivity to shifts in non-expert weightage. Furthermore, the relative priority rankings of chemicals remained entirely unchanged across all tested weight combinations, as illustrated in Table 1. For instance, high-priority substances such as (Lead, Mercury, etc.) retained their positions regardless of whether non-expert weights were set to 5% or 50%. This consistency highlights the stability of our original 30% weighting approach.

However, deviations exceeding 10% were observed for a small subset of chemicals, including the Aniline family, Di-isocyanates, Chromium VI, and Perfluorinated and Polyfluorinated Substances (PFAS). These discrepancies arose from notable differences in how experts and non-experts scored their concerns. For example, non-experts perceived PFAS as a higher-risk chemical compared to experts, leading to greater variability in scores as non-expert weightage increased.

Overall, the analysis confirms that our methodology is resilient to fluctuations in non-expert weightage, validating the appropriateness of the 30% weighting used in this study. Nevertheless, the observed deviations for specific chemicals emphasise the importance of incorporating sensitivity analyses into future iterations of prioritisation workflows.

Supplementary Material S3: HBM Programme Review

Serial no.	Initiative/ Programme	Country/ Region	Target Chemicals	Key Objectives	Reference
1	HBM4EU	EU-wide	Heavy metals (Cd, Pb, Hg), PFAS, Phthalates, Bisphenols, PAHs, etc.	Funded under the Horizon 2020 framework, HBM4EU brought together 30 countries, the European Environment Agency (EEA), and the European Commission and played a key role in coordinating and advancing human biomonitoring (HBM) in Europe.	[1, 2]
2	PARC	EU-wide	Heavy metals, Phthalates, Cotinine, Bisphenols, DINCH, PFAS, Neonicotinoids, Pyrethroids, Organophosphates, Glyphosate, AMPA, heavy metals, cotinine, etc.	PARC advances chemical risk assessment, supporting the EU's sustainability and "Zero Pollution" goals.	[3, 4]
3	FLEHS (Flanders Environment and Health Study)	Belgium	Heavy metals, endocrine disruptors, perfluorinated compounds, phthalates, flame retardants, VOC (benzene) etc.	The FLEHS is a long-standing HBM programme in Flanders, Belgium, aimed at assessing exposure to environmental pollutants and their potential health effects on the population. Initiated in 2002, FLEHS is a key component of the Flemish HBM Programme, providing valuable data for environmental policy and public health interventions.	[5, 6, 7]
4	GerES (German Environmental Survey)	Germany	Flame retardant chemicals, heavy metals, aprotic solvents, UV filters, PFAS, Persistent organic pollutants, phthalates and hexamoll (DINCH), pesticides, etc.	The German Environmental Survey (GerES) is a population representative national HBM and environmental exposure assessment programme conducted by the German Environment Agency (Umweltbundesamt, UBA). Since its inception in 1985, GerES has been one of the most comprehensive long-term environmental health studies in Europe.	[8, 9]
5	Esteban Study	France	Metals, Benzene, Phthalates, Bisphenol A, Perfluorochemicals, VOCs, PCB non-dioxin like Polybrominated compounds, Pesticides, PAH, Glycol ethers, Organotins, Parabens, VOCs, Cotinine and Mycotoxins.	The ESTEBAN Study (Étude de Santé sur l'Environnement, la Biosurveillance, l'Activité physique et la Nutrition) is a national HBM programme in France, conducted by the French Public Health Agency. Launched in 2014, ESTEBAN builds upon previous national surveys, such as the ENNS (National Nutrition and Health Survey), to evaluate the exposure of the French population to environmental pollutants and their potential health effects.	[10, 11]
6	MoBa (Mother and Child Cohort Study)	Norway	Pollutants/contaminants (Pesticides, acrylamide, PCBS, Bisphenols etc.) Lifestyle (smoking, per- and polyfluoroalkyl substances (PFAS)), polychlorinated biphenyls (PCBs), pesticides, flame retardants, phthalates, bisphenols, parabens, acrylamide, heavy metals.	Run by the Norweigan institute of public health. The human biomonitoring programme is part of the MOBA study.	[12]

Serial no.	Initiative/ Programme	Country/ Region	Target Chemicals	Key Objectives	Reference
7	Slovenia National Human Biomonitoring	Slovenia	Heavy metals (As, Cd, Cu, Hg, Se, Mn), POPs (PCBs, DDTs, etc), environmental phenols, phthalates, and DINCH, PAHs, glyphosate and AMPA, Bisphenols, parabens, Triclosan, PFASs.	Slovenia's HBM Programme, launched in 2007, assesses population exposure to chemicals and related health risks, aligning with EU projects like HBM4EU. Initiated in 2006 by the National Chemicals Bureau, which oversees coordination and funding under the Slovenian Chemicals Act, the National Institute of Public Health (NIPH) developed the programme proposal.	[13, 14, 15, 16, 17, 18, 19]
8	National Health and Nutrition Examination Survey (NHANES)	United States	Adducts, Tobacco Alkaloids and Metabolites, Disinfection By-Products, Flame Retardant Metabolites, Organophosphorus Insecticides: Dialkyl Phosphate Metabolites, Metals and Metalloids, Perchlorate and Other Anions, Perfluoroalkyl and Polyfluoroalkyl Substances: Surfactants, Phthalate and Phthalate Alternative Metabolites, Volatile Organic Compounds (VOCs), VOC Metabolites.	The U.S. Human Biomonitoring Programme, conducted through NHANES, assesses population health, nutrition, and chemical exposure. Established in the 1960s by the National Center for Health Statistics under the CDC, NHANES has operated continuously since 1999. It employs a nationally representative sampling of non-institutionalised civilians of all ages, combining self-reported data with physical examinations and laboratory tests.	[20]
9	Canadian Health Measures Survey (CHMS)	Canada	Flame retardants, Organochlorines, Polychlorinated biphenyls, Chlorophenols, Per- and polyfluoroalkyl substances, Plasticizers, Polycyclic aromatic hydrocarbons, Acrylamide, Volatile organic compounds, Metals and trace elements, Self-care and consumer product chemicals, Nicotine, Pesticides.	Canada's Human Biomonitoring Programme has been integrated into the Canadian Health Measures Survey (CHMS) since 2007. Led by Statistics Canada in collaboration with Health Canada and the Public Health Agency of Canada, CHMS collects biological samples from a representative population to track trends, evaluate health risks, and inform policies.	[21]
10	China National Human Biomonitoring Programme	China	Inorganic chemicals (13 compounds), Poly- and per-fluorinated alkyl substances (PFAS) (17 compounds), Phthalate alternative metabolites in urine (11 compounds), Metabolites of polycyclic aromatic hydrocarbon (PAHs) in urine: (9 compounds), Environmental phenol alternative in urine: (4 compounds), Benzene metabolites in urine (2 compounds).	The China National Human Biomonitoring (CNHBM) programme, launched by the National Institution of Environmental Health (NIEH) of China CDC in 2017–2018, is a population-representative study assessing environmental chemical exposure in the Chinese population. It includes children and enables longitudinal follow-up.	[22]

Serial no.	Initiative/ Programme	Country/ Region	Target Chemicals	Key Objectives	Reference
11	Swedish Environmental Monitoring Programme (SMP)	Sweden	Elements (e.g., heavy metals), Chlorinated, brominated and fluorinated persistent organic pollutants (POPs), Phthalates, Phthalate alternatives, Bisphenols, Phosphorous flame retardants, Polyaromatic hydrocarbons (PAH), Pesticides, Biocides/preservatives, UV-filters.	The Swedish Human Biomonitoring (HBM) programme, part of the Health-Related Environmental Monitoring Program (HÄMI) overseen by the Swedish Environmental Protection Agency, tracks the population's exposure to environmental chemicals, focusing on vulnerable groups like children and women to identify trends and health risks.	[23, 24]
12	Czech Republic HBM Programme	Czech Republic	Toxic metals (cadmium, lead, mercury) Organochlorinated pesticides (metabolites of DDT, hexachlorobenzene, hexachlorocyclohexane) polychlorinated biphenyls (PCBs) per- and polyfluorinated compounds (PFAS), Flame retardants, Phthalate metabolites, Bisphenols, as well as beneficial elements and vitamins (e.g. selenium, iodine, vitamin D).	The Czech Republic's Human Biomonitoring (HBM) programme, running since 1994, assesses chemical exposure in adults, children, and breastfeeding mothers to evaluate health risks. It highlights early-life exposure to a wide range of chemicals, reflecting trends in the developed world.	[25]
13	Korean National Environmental Health Survey (KoNEHS)	South Korea	Heavy metals, Polycyclic aromatic hydrocarbon (PAHs), Environmental Phenols, Phthalate metabolites, Pyrethroid pesticides, Volatile Organic Compounds (VOCs), Per- and polyfluoroalkyl substances (PFAS), butylparaben, benzophenone-3, PCBs, Organochlorine pesticides, Polybrominated Diphenyl Ethers, PBDEs, Cotinine.	The Korean National Human Biomonitoring (KNHBM) programme was launched in 2005 as part of the National Health and Nutrition Survey to assess environmental pollutant exposure in the general population.	[26, 27]
14	New Zealand biological monitoring programme	New Zealand	Heavy metals (mercury, arsenic, cadmium, chromium, thallium, antimony), phenols, phthalate metabolites, cotinine, parabens and fluoride, UV filters (Benzophenone-3), Triclosan.	The New Zealand Ministry of Health commissioned the programme in 2012, with the Centre for Public Health Research (CPHR) at Massey University overseeing its implementation. Two analytical laboratories were subcontracted to carry out the laboratory analyses of blood and urine samples.	[28]

References:

1. HBM4EU. HBM4EU substances. Retrieved from: https://www.hbm4eu.eu/hbm4eu-substances/ Accessed on: 17th June 2024
2. Ougier E, Ganzleben C, Lecoq P, Bessems J, David M, Schoeters G, Lange R, Meslin M, Uhl M, Kolossa-Gehring M, Rousselle C. Chemical prioritisation strategy in the European human biomonitoring initiative (HBM4EU)–development and results. International Journal of Hygiene and Environmental Health. 2021 Jul 1;236:113778. https://doi.org/10.1016/j.ijheh.2021.113778.
3. European Partnership for the Assessment of Risks from Chemicals (PARC), What we do. Retrieved from: https://www.eu-parc.eu/what-we-do Accessed on: 17th June 2024
4. Partnership for the Assessment of Risks from Chemicals (PARC). PARC T4.1.2.: Selected biomarkers of exposure for PARC Aligned Studies.
5. What do we measure? (factsheets), Centre for Environment and Health, Flanders. Retrieved from: https://www.omgeving-en-gezondheid.be/nl/onderzoek/wat-meten-we-factsheets#paddle_components_text_block_11ce459a-c8cb-45aa-b273-b22b95bf116f Accessed on: 19th July 2024
6. Schoeters G, Den Hond E, Colles A, Loots I, Morrens B, Keune H, Bruckers L, Nawrot T, Sioen I, De Coster S, Van Larebeke N. Concept of the Flemish human biomonitoring programme. International journal of hygiene and environmental health. 2012 Feb 1;215(2):102-8. https://doi.org/10.1016/j.ijheh.2011.11.006
7. Schoeters G, Govarts E, Bruckers L, Den Hond E, Nelen V, De Henauw S, Sioen I, Nawrot TS, Plusquin M, Vriens A, Covaci A. Three cycles of human biomonitoring in Flanders– Time trends observed in the Flemish Environment and Health Study. International journal of hygiene and environmental health. 2017 Mar 1;220(2):36-45. https://doi.org/10.1016/j.ijheh.2016.11.006
8. Umweltbundesamt. Cooperation for the promotion of human biomonitoring. Retrieved from: https://www.umweltbundesamt.de/en/topics/health/assessing-environmentally-related-health-risks/human-biomonitoring/cooperation-for-the-promotion-of-human#background-objectives-and-tasks-of-cooperation. Accessed on: 17th July 2024
9. Umweltbundesamt. Human biomonitoring commission (HBM commission). Retrieved from: https://www.umweltbundesamt.de/en/topics/health/commissions-working-groups/human-biomonitoring-commission-hbm-commission. Accessed on: 11th July 2024
10. Fillol C, Vandentorren S. National human biomonitoring programme in France: Selection of substances and prioritisation of biomarkers. French Institute for Public Health Surveillance (InVS), Department of Environmental Health. Retrieved from: https://www.umweltbundesamt.de/sites/default/files/medien/378/dokumente/clemence_fillol_national_human_biomonitoring_programme_in_france_selection_of_substances_and_prioritization_of_biomarkers.pdf Accessed on: 9th June 2024.
11. Dereumeaux C, Fillol C, Charles MA, Denys S. The French human biomonitoring program: First lessons from the perinatal component and future needs. International Journal of Hygiene and Environmental Health. 2017 Mar 1;220(2):64-70. https://doi.org/10.1016/j.ijheh.2016.11.005.
12. Norwegian Institute of Public Health. Research and data access. Norwegian Institute of Public Health. Retrieved from: https://www.fhi.no/en/ch/studies/moba/for-forskere-artikler/research-and-data-access/. Accessed on: 12th April 2024.
13. Runkel AA, Križanec B, Lipičar E, Baskar M, Hrženjak V, Kodba ZC, Kononenko L, Kanduč T, Mazej D, Tratnik JS, Horvat M. Organohalogens: A persisting burden in Slovenia?. Environmental Resear□h. 2021 Jul 1;198. https://doi.org/10.1016/j.envres.2021.111224
14. Tratnik JS, Falnoga I, Mazej D, Kocman D, Fajon V, Jagodic M, Stajnko A, Trdin A, Šlejkovec Z, Jeran Z, Osredkar J. Results of the first national human biomonitoring in Slovenia: Trace elements in men and lactating women, predictors of exposure and reference values. International journal of hygiene and environmental health. 2019 Apr 1;222(3):563-82. https://doi.org/10.1016/j.ijheh.2019.02.008

15. Runkel AA, Mazej D, Tratnik JS, Tkalec Ž, Kosjek T, Horvat M. Exposure of men and lactating women to environmental phenols, phthalates, and DINCH. Chemosphere. 2022 Jan 1;286. https://doi.org/10.1016/j.chemosphere.2021.131858.
16. Joksić AŠ, Tratnik JS, Mazej D, Kocman D, Stajnko A, Eržen I, Horvat M. Polycyclic aromatic hydrocarbons (PAHs) in men and lactating women in Slovenia: results of the first national human biomonitoring. International Journal of Hygiene and Environmental Health. 2022 Apr 1;241. https://doi.org/10.1016/j.ijheh.2022.113943.
17. Stajnko A, Tratnik JS, Kosjek T, Mazej D, Jagodic M, Eržen I, Horvat M. Seasonal glyphosate and AMPA levels in urine of children and adolescents living in rural regions of Northeastern Slovenia. Environment International. 2020 O□t 1;143. https://doi.org/10.1016/j.envint.2020.105985.
18. Tkalec Ž, Kosjek T, Tratnik JS, Stajnko A, Runkel AA, Sykiotou M, Mazej D, Horvat M. Exposure of Slovenian children and adolescents to bisphenols, parabens and Triclosan: urinary levels, exposure patterns, determinants of exposure and susceptibility. Environment International. 2021 Jan 1;146. https://doi.org/10.1016/j.envint.2020.106172.
19. Runkel AA, Stajnko A, Tratnik JS, Mazej D, Horvat M, Přibylová P, Kosjek T. Exposure of children and adolescents from Northeastern Slovenia to per-and polyfluoroalkyl substances. Chemosphere. 2023 Apr 1;321. https://doi.org/10.1016/j.chemosphere.2023.138096.
20. Centers for Disease Control and Prevention. Biomarker groups: Reported in CDC's National Report on Human Exposure to Environmental Chemicals. U.S. Department of Health and Human Services. Retrieved from: https://www.cdc.gov/environmental-exposure-report/media/pdfs/Biomarker-Groups-Infographic-508.pdf Accessed on 10th March, 2024.
21. Health Canada. Sixth report and fact sheets on human biomonitoring of environmental chemicals in Canada: Canadian Health Measures Survey [Internet]. National Biomonitoring Section, Environmental Health Science and Research Bureau, Healthy Environments and Consumer Safety Bran□h; 2021 De□14. Retrieved from: https://resilient-health.ca/wp-content/uploads/2021/12/CHMS_Cycle6_Release_Presentation_EN_Final.pdf. Accessed on 19th March 2024.
22. Cao Z, Lin S, Zhao F, Lv Y, Qu Y, Hu X, Yu S, Song S, Lu Y, Yan H, Liu Y. Cohort profile: China National Human Biomonitoring (CNHBM)—a nationally representative, prospective □ohort in Chinese population. Environment international. 2021 Jan 1;146. https://doi.org/10.1016/j.envint.2020.106252.
23. Pineda S, Lignell S, Gyllenhammar I, Lampa E, Benskin JP, Lundh T, Lindh C, Kiviranta H, Glynn A. Exposure of Swedish adolescents to elements, persistent organic pollutants (POPs), and rapidly excreted substances–the Riksmaten adolescents 2016-17 national survey. International journal of hygiene and environmental health. 2023 Jun 1;251. https://doi.org/10.1016/j.ijheh.2023.114196.
24. HBM4EU, Group 3 National Hub Template (HBM data for policy development), Retrieved from: https://www.hbm4eu.eu/wp-content/uploads/2022/07/SWEDEN.pdf. Accessed on 18th August 2024.
25. National Institute of Public Health, Czech Republic. Results of human biomonitoring. Retrieved from: https://szu.gov.cz/temata-zdravi-a-bezpecnosti/zivotni-prostredi/biologicky-monitoring/vysledky-lidskeho-biomonitoringu/. Accessed on 9th May 2024.
26. Hong S, Kim OJ, Jung SK, Jeon HL, Kim S, Kil J. The Exposure Status of Environmental Chemicals in South Korea: The Korean National Environmental Health Survey 2018–2020. Toxi□s. 2024 Nov 19;12(11):829. https://doi.org/10.3390/toxics12110829.
27. Son, J. Y., Lee, J., Paek, D., & Lee, J. T. (2009). Blood levels of lead, cadmium, and mercury in the Korean population: results from the Second Korean National Human Exposure and Bio-monitoring Examination. Environmental Research, 109(6), 738-744. https://doi.org/10.1016/j.envres.2009.03.012.
28. Mannetje A, Coakley J, Douwes J. Report on the biological monitoring of selected chemicals of concern. Results of the New Zealand biological monitoring programme, 2014-2016. Retrieved from: https://publichealth.massey.ac.nz/assets/Uploads/SOCs-Report-FINAL-06032018.pdf. Accessed on 9th September 2024.
